# Metronomic vinorelbine: Anti-angiogenic activity *in vitro* in normoxic and severe hypoxic conditions, and severe hypoxia-induced resistance to its anti-proliferative effect with reversal by Akt inhibition

**DOI:** 10.3892/ijo.2015.3059

**Published:** 2015-06-19

**Authors:** L. MAVROEIDIS, H. SHELDON, E. BRIASOULIS, M. MARSELOS, P. PAPPAS, A.L. HARRIS

**Affiliations:** 1 Molecular Oncology Laboratories, Weatherall Institute of Molecular Medicine, University of Oxford, John Radcliffe Hospital, Oxford OX3 9DS, UK; 2 Department of Hematology, School of Medicine, University of Ioannina, University Campus, 45110 Ioannina, Greece; 3 Interscience Molecular Oncology Laboratory, Cancer Biobank Center, University of Ioannina, University Campus, 45110 Ioannina, Greece; 4 Department of Pharmacology, School of Medicine, University of Ioannina, University Campus, 45110 Ioannina, Greece

**Keywords:** metronomic chemotherapy, vinorelbine, anti-angiogenic, hypoxia, resistance to anti-angiogenic therapy

## Abstract

Metronomic chemotherapy is the protracted, dense administration of low sub-toxic doses of chemotherapy, to inhibit tumor angiogenesis. Vinorelbine is an orally bioavailable vinca alkaloid shown to be useable for metronomic administration. In clinical trials, metronomic vinorelbine has been demonstrated to generate sustainable antitumor efficacy at low nanomolar (nM) concentrations with negligible toxicity. We sought to determine whether the clinically relevant metronomic concentration of vinorelbine is anti-angiogenic *in vitro* and whether hypoxia, often induced by anti-angiogenic therapy, modifies its effectiveness. We found that the metronomic concentration of 10 nM vinorelbine inhibited human umbilical vein endothelial cell (HUVEC) proliferation, migration, tube formation and sprouting. Severe hypoxia, did not affect the inhibitory effect of metronomic vinorelbine on migration, tube formation and sprouting. However, severe hypoxia reduced its anti-proliferative effect by decreasing its ability to induce G2/M block as it shifted the cell population to the G1 phase and decreased the fraction of the cells in the DNA synthesis S phase. Furthermore, the pro-apoptotic effects of 10 nM vinorelbine were also decreased. Metronomic vinorelbine decreased the Bcl-2/Bax ratio in normoxia whereas the ratio was reduced in severe hypoxia but unaltered by vinorelbine treatment. Akt signals to an anti-apoptotic pathway and we demonstrated that the Akt inhibitor V reversed the protective effect of severe hypoxia. Thus, we provide evidence for the anti-angiogenic basis of metronomic vinorelbine and we show that severe hypoxia mediates resistance to its anti-proliferative effect on endothelial cells. Akt warrants further investigation as a potential target to circumvent this hypoxic resistance.

## Introduction

Metronomic chemotherapy is the chronic administration of low dose chemotherapy as opposed to the conventional chemotherapy protocol of the pulsatile administration of a maximum tolerated dose (1). It is a promising protocol of chemotherapy with a low toxic profile and encouraging results in certain clinical trials (2). Metronomic chemotherapy seems to have pleiotropic effects (2). It can target the cancer cell and modulate the immune system but it is primarily considered anti-angiogenic (2). Antimitotics, including taxanes and vinca alkaloids are lead drugs for metronomic treatment as they inhibit angiogenesis through multiple mechanisms (3).

Vinorelbine is a semisynthetic vinca alkaloid with the additional advantage of the oral formulation which favors its use in the chronic administration protocol of metronomic chemotherapy (4). Briasoulis *et al* demonstrated that the metronomic administration of vinorelbine, given three times a week, maintains low nanomolar steady state concentrations in the blood (4) and yields objective responses of prolonged duration with negligible toxicity (4,5). The authors suggested that the antitumor efficacy is likely due to anti-angiogenic action because of the profile of circulating angiogenic biomarkers in responding patients, the low nanomolar concentrations of the drug and the minimal toxicity (5).

Unfortunately, anti-angiogenic therapies have only an ephemeral effect (6), since after the initial response resistance develops leading to treatment failure (6). Tumors acquire resistance to VEGF-targeted agents through activation of different modes of vascularization, upregulation of alternative pro-angiogenic signaling pathways and recruitment of pro-angiogenic cells (6–8). Anti-angiogenic treatment cut off the tumor blood supply creating a hypoxic microenvironment. Treatment-induced hypoxia is shown to be the initiating factor of this secondary resistance to anti-VEGF therapies, as reviewed by Loges *et al* (8). Furthermore, hypoxia is associated with resistance to chemotherapy (9–11). Hypoxia modulates the intrinsic apoptotic pathway and alters cell cycle leading to refractoriness to cell cycle specific agents (9).

Drugs with vascular disrupting properties, such as microtubule targeting agents, can rapidly promote and sustain conditions of severe hypoxia with partial oxygen pressure <2.5 mm Hg in the tumor center (12,13). Considering the fact that hypoxia is the triggering factor of the evasive resistance to certain anti-angiogenic therapies (6–8) and it confers resistance to chemotherapy (9–11), we questioned whether severe hypoxia can mediate resistance to the anti-angiogenic action of metronomic vinorelbine.

The rational combination of metronomic chemotherapy with a targeted agent can enhance the efficacy of metronomic treatment (14). The Akt pathway is a critical modulator of angiogenesis and cell survival (15). Both vinca alkaloids and Akt converge to the intrinsic mitochondrial apoptotic pathway to regulate cell death (16,17). We tested whether Akt inhibition could sensitize endothelial cells to the anti-proliferative action of metronomic vinorelbine.

In this study, we sought to determine whether the clinically relevant metronomic concentration (5) of 10 nM is anti-angiogenic *in vitro* and we compared it with the concentration of 1 μM which simulates the peak plasma levels of the conventional chemotherapy protocol (18). We show that 10 nM vinorelbine inhibits the sequential steps of sprouting angiogenesis (19) such as migration, tube formation and proliferation. We found that severe hypoxia (0.1% O_2_) confers resistance to the anti-proliferative action of metronomic vinorelbine due to G1 arrest and attenuation of apoptosis. The Bcl-2 protein family is implicated in the cell death caused by the microtubule targeting agents (MTAs) (16) and we questioned whether Bcl-2 is also regulated by severe hypoxia. Finally, we sought to find a way to circumvent this hypoxic resistance and we report that combination with Akt inhibition sensitizes HUVECs to the action of 10 nM vinorelbine.

## Materials and methods

### Cell culture and chemical compounds

Human umbilical vein endothelial cells (HUVECs), supplied from Lonza, were cultured on culture dishes (Corning) coated with gelatin (0.1% w/v) and were fed with endothelial basal media supplemented with growth factors (EGM-2; Lonza). Incubation in severe hypoxia (0.1% O_2_) was undertaken in an invivo2 400 hypoxic workstation (Ruskin Technologies). Vinorelbine (Tocris Bioscience) was dissolved in dimethyl sulfoxide and used at the indicated concentrations. Akt inhibitor V (Calbiochem) was dissolved in dimethyl sulfoxide and used at a concentration of 10 μM.

### Immunoblotting

Cells were lysed with RIPA buffer (Sigma-Aldrich), supplemented with a cocktail of protease and phosphatase inhibitors (Roche), by incubating on ice for 20 min. Cell lysate was clarified by centrifugation for 10 min at 4°C and protein was quantified with Bio-Rad protein assay. We probed for Bcl-2 (Santa Cruz, sc-509), Bax (Santa Cruz, sc-493), p27 Kip (Cell Signaling, no. 2552) and β-actin as loading control (anti-β-actin HRP conjugate, Sigma-Aldrich, A3854). Quantification of the intensity of the protein bands was performed with ImageJ.

### Proliferation assay

Proliferation was determined with the CyQUANT^®^ assay (Life Technologies). Cells were plated on 96-well plates in a density of 2,000 cells/well and treated as appropriate. Afterwards the media were aspirated and the reagent was added according to the manufacturer’s protocol for 1 h. Fluorescence was measured with SpectraMax M2 multimode microplate reader. Proliferation was assessed by the relative fluorescent unit (RFU) normalized to the untreated control.

### Wound healing assay

Migration was assessed with the wound healing assay. One hundred thousand cells/well were seeded on a 24-well plate supplied by Essen BioScience. When the cell monolayer became confluent, a scratch wound was performed at the time-point 0 (t: 0). An image of the wound was captured at t: 0 and the cells were subsequently treated with vinorelbine in normoxia or severe hypoxia for 6 h (t: 6 h). At t: 6 h a second image was taken. Images of the wound were captured by the IncuCyte (Essen BioScience), analyzed with the built in algorithm and quantified by assessing the wound confluence parameter. The wound confluence value at t: 6 h was corrected by subtracting the initial wound confluence at t: 0.

### Matrigel assay

Tube formation was assessed with the Matrigel assay. 96-well plates were coated with 50 μl Matrigel (BD Matrigel™ basement membrane matrix) which was allowed to set for 30 min in 37°C. Fifteen thousand HUVECs/well were subsequently seeded on the top of the matrix and the cells were treated with vinorelbine in normoxia or severe hypoxia for 6 h. Phase contrast images were taken with the EVOS Cell Imaging System (Life Technologies) at the end of the treatment. Tube formation was determined by the number of polygones of the tube network.

### Hanging drop assay

Angiogenic sprouting was examined with the hanging drop assay. Briefly, drops of HUVEC suspension with 750 cells/20 μl were dispensed on the inner side of an inverted culture dish lid. The lid was carefully placed back on the top of the dish and the droplets were allowed to form spheroids overnight. The spheroids were then pelleted, resuspended in fibrin solution (2 mg/ml) containing 0.15 U/ml aprotinin and dispensed in 24-well plates containing thrombin. The fibrin solution with HUVEC spheroids was mixed gently with thrombin (0.625 U/ml) and allowed to clot for 20 min in 37°C. Media with or without vinorelbine were then added in the well on the top of the clot. HUVEC spheroids were treated in normoxia or severe hypoxia for 24 h. Phase contrast images were taken at the end of the treatment with the EVOS Cell Imaging System (Life Technologies). Sprouting was determined by quantifying the area occupied by the sprout outgrowth at the end of the treatment. Quantification of the sprout area was performed with ImageJ.

### Cell cycle analysis

Cells were treated for 24 h in normoxia or severe hypoxia and were subsequently harvested, washed with PBS and fixed with cold 70% ethanol overnight at 4°C. Afterwards, the cells were washed twice with PBS and then treated with a solution containing ribonuclease I (20 μg/ml) and propidium iodine (PI) (100 μg/ml) at room temperature. After incubation for 15 min the cells were analyzed in a FACS Analyzer CyAn ADP on FL3 channel. Quantification was done with FlowJo software v.10 by employing the built-in algorithm.

### Analysis of apoptosis

Cells were treated as appropriate and were subsequently harvested and washed with PBS. Cells were resuspended in binding buffer to bring 10^5^ cells/100 μl and incubated with PI in the final concentration of 1 μg/ml and Annexin V conjugated with AlexaFluor 647 in the final dilution of 1/100. After incubation for 15 min at room temperature, stained cells were analyzed on FL3 channel for PI and FL8 for Annexin V by using a FACS Analyzer CyAn ADP. Reagents were supplied by Molecular ProbesR.

### Statistical analyses

Statistical analyses and graphs were performed with GraphPad prism v.5. Statistical comparisons were carried out by using one-way ANOVA or un-paired t-test.

## Results

### Dose- and time-dependent effect of vinorelbine on endothelial cell proliferation

**Figure 1:**
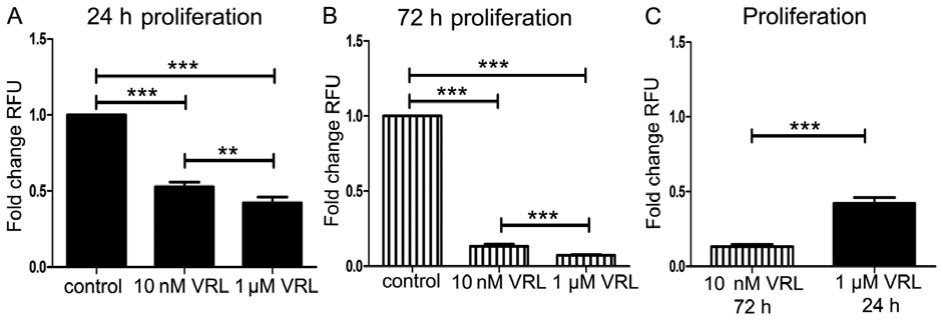
The effect of vinorelbine (VRL) on HUVEC proliferation as determined by the CyQUANT assay. (A) The effect at 24-h treatment (24 h). (B) The effect at 72-h treatment (72 h). (C) VRL (10 nM) for 72 h is more potent in inhibiting proliferation than 1 μM for 24 h. Proliferation (y-axis) is assessed by the relative fluorescent unit (RFU) normalized to the corresponding control in either 24 or 72 h. Results are expressed as mean ± SD of three independent experiments. Error bars depict standard deviation (SD). (A and B) One-way ANOVA; (C) un-paired t-test; ^**^P<0.01; ^***^P<0.001.

To investigate whether metronomic vinorelbine is anti-angiogenic, we first tested the effect on endothelial cell proliferation which is one of the sequential steps of sprouting angiogenesis (19). We compared the effect of 10 nM, a clinically relevant metronomic concentration (4,5) with the effect of 1 μM, which is close to the transient peak plasma levels of the drug in maximum tolerated dose chemotherapy (18). Ten nanomolar inhibited proliferation by 47% (P<0.001) and 1 μM by 58% (P<0.001) at 24-h treatment (Fig. 1A). Ten nanomolar inhibited proliferation by 87% (P<0.001) and 1 μM by 93% (P<0.001) at 72-h treatment (Fig. 1B). Vinorelbine inhibited proliferation in a dose responsive manner but the metronomic concentration of 10 nM was more effective at 72 h than the concentration of 1 μM at 24 h (Fig. 1C). The latter indicates the favorable effect of the prolonged metronomic treatment, compared to the short-term treatment resembling conventional chemotherapy.

### The metronomic concentration of 10 nM vinorelbine inhibits migration, tube formation and sprouting without affecting cell viability

To further examine the anti-angiogenic action of metronomic vinorelbine, we investigated the effect of 10 nM on migration, tube formation and sprouting *in vitro* and we compared it with the concentration of 1 μM (18).

We assessed migration and tube formation in the short-term treatment of 6 h to avoid the interference from the anti-proliferative effect of metronomic vinorelbine.

**Figure 2:**
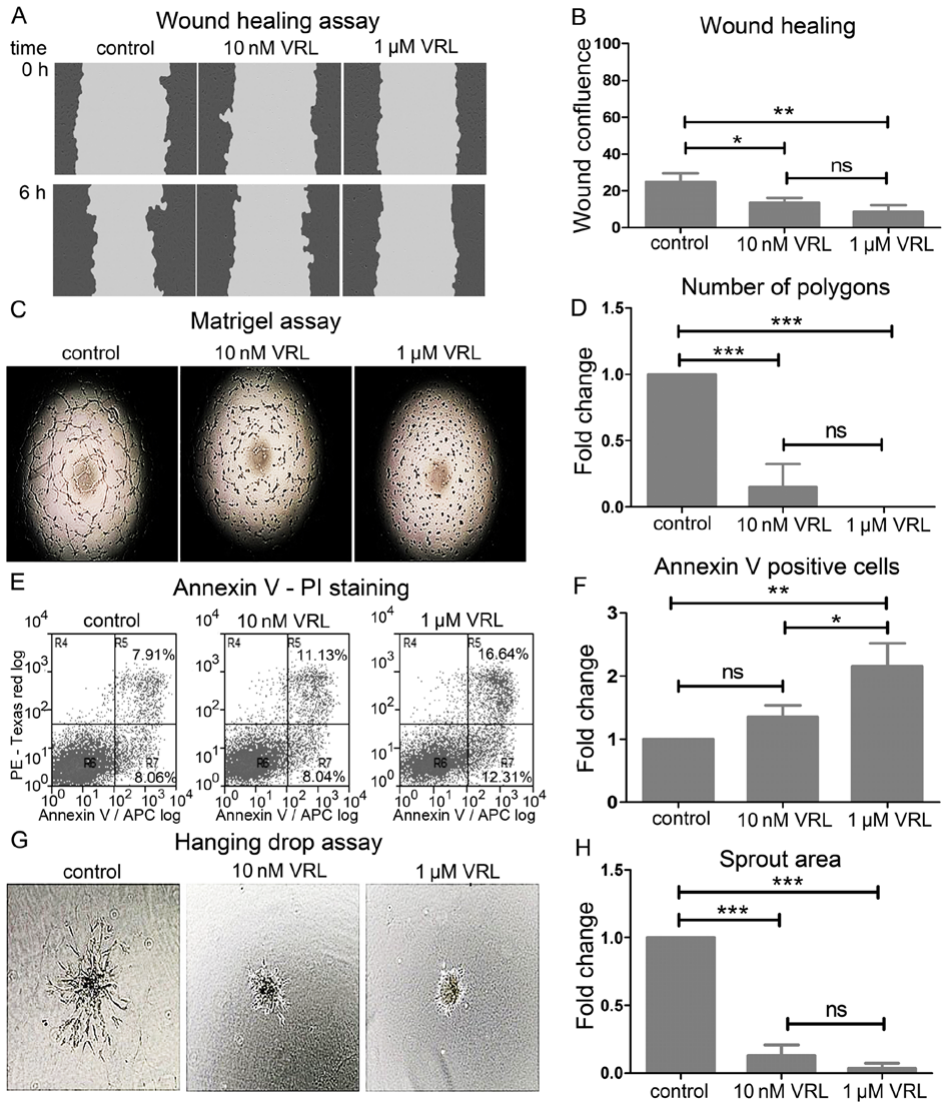
The effect of vinorelbine (VRL) on migration, tube formation, viability and sprouting of HUVECs. (A and B) Migration in the wound healing assay. (A) Representative images showing the scratch wound mask at the time-points 0 and 6 h (hours). Note the delay of the healing process with VRL at 6 h compared to control. (B) The effect of VRL on wound healing as determined by the wound confluence parameter. Results are expressed as mean wound confluence ± SD of three independent experiments. Error bars depict standard deviation. One-way ANOVA; ns, not significant; ^*^P<0.05; ^**^P<0.01. (C and D) Tube formation in the Matrigel™ assay. (C) Representative images showing suppressed tube formation upon treatment with VRL. (D) The effect of VRL on tube formation as determined by the number of polygons formed. (E and F) Annexin V-PI staining analysis with FACS after 6-h treatment with VRL. (E) Representative dual parametric dot plot showing staining for Annexin V and/or PI. The values represent percentages of the total cell number. R4, PI-positive (necrotic cells); R5, Annexin V + PI-positive (late apoptotic cells); R6, negative (viable cells); R7, Annexin V-positive (early apoptotic cells). R5+R7, total apoptotic cells. (F) The effect of VRL on cell death as determined by the Annexin V-positive cells. (G and H) Angiogenic sprouting in the hanging drop assay. (G) Representative images showing inhibition of the sprout outgrowth upon treatment with VRL. (H) The effect of VRL on sprouting as determined by the spout area. (D, F and H) Results are expressed as mean fold change ± SD of three independent experiments. Error bars represent standard deviation (SD). One-way ANOVA; ns, not significant; ^*^P<0.05; ^**^P<0.01; ^***^P<0.001.

Vinorelbine inhibited migration as determined by the wound healing assay (Fig. 2A). Ten nanomolar vinorelbine decreased the wound confluence by 1.8 times after the scratch wound while 1 μM decreased it by 2.81 times (Fig. 2B).

Vinorelbine inhibited tube formation as determined by the Matrigel assay (Fig. 2C). Ten nanomolar vinorelbine decreased the number of polygons formed within the tube network by 85% (P<0.001) 6 h after plating HUVECs on Matrigel while 1 μM completely prevented the formation of the tube network (P<0.001) (Fig. 2D).

The inhibition of the functions above by 10 nM vinorelbine was not attributed to cell toxicity (Fig. 2E). Ten nanomolar vinorelbine did not change significantly the cell percentage stained positive for Annexin V at 6 h whereas 1 μM vinorelbine increased it by 2.16-fold (P<0.01) (Fig. 2F).

Finally, we examined the overall effect on sprouting angiogenesis with the hanging drop assay (Fig. 2G). Sprouting angiogenesis involves a sequence of events starting with endothelial sprouting into tip cells, tip cell migration, stalk cell proliferation, branching and finally lumen formation (19). We assessed sprouting in fibrin gel at 24 h. Ten nanomolar decreased the area of the sprout outgrowth by 87% (P<0.001) while 1 μM almost completely disrupted sprouting (P<0.001) (Fig. 2H).

### Severe hypoxia does not interfere with the inhibitory action of 10 nM vinorelbine on the endothelial cell migration, tube formation or sprouting

**Figure 3:**
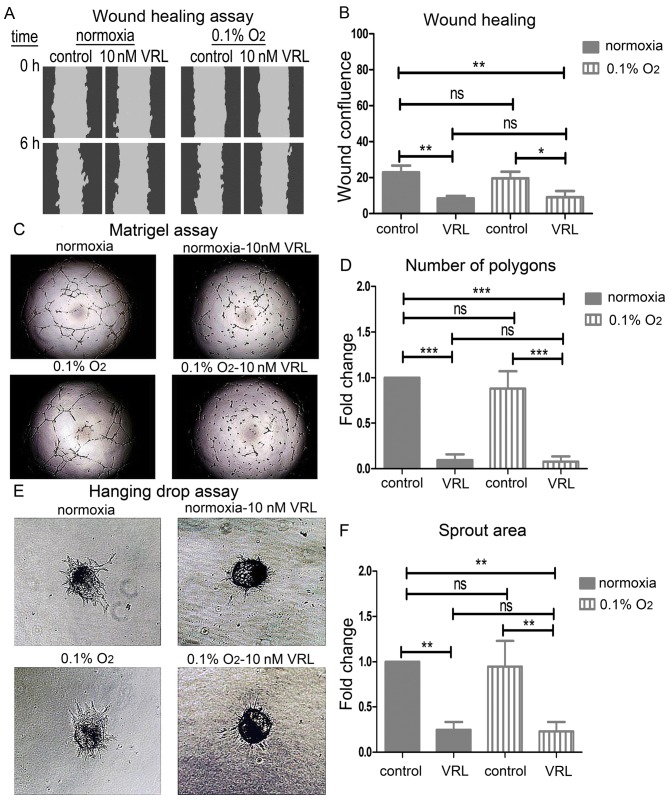
The effect of severe hypoxia (0.1% O_2_) on migration, tube formation and sprouting of HUVECs treated with 10 nM vinorelbine (VRL). (A and B) Migration in the wound healing assay. (A) Representative images showing the scratch wound mask at the time-points 0 and 6 h (hours). Note the delay of the healing process with VRL at 6 h both in normoxia and severe hypoxia. (B) The effect of VRL and severe hypoxia on wound healing as determined by the wound confluence parameter. Results are expressed as the mean wound confluence ± SD of three independent experiments; error bars represent standard deviation (SD), one-way ANOVA; ns, not significant; ^*^P<0.05; ^**^P<0.01. (C and D) Tube formation in the Matrigel™ assay. (C) Representative images showing suppressed tube formation in normoxia and severe hypoxia upon treatment with VRL. (D) The effect of VRL and severe hypoxia on tube formation as determined by the number of polygons formed by HUVECs. (E and F) Angiogenic sprouting in the hanging drop assay. (E) Representative images showing impediment of the sprout outgrowth both in normoxia and severe hypoxia upon treatment with VRL. (F) The effect of VRL and severe hypoxia on sprouting as determined by the sprout area. (D and F) Results are expressed as mean fold change ± SD of three independent experiments. Error bars represent standard deviation (SD). One-way ANOVA; ns, not significant; ^**^P<0.01; ^***^P<0.001.

Having shown that metronomic vinorelbine is anti-angiogenic and considering that hypoxia, which is exacerbated by anti-angiogenic therapy, is associated with treatment failure (8), we investigated whether severe hypoxia (0.1% O_2_) mediates resistance to metronomic vinorelbine treatment. We show that the metronomic concentration of 10 nM vinorelbine reduced the wound confluence to the same extent (Fig. 3B) in normoxia and severe hypoxia in the wound healing assay (Fig. 3A). Moreover, it reduced the number of polygons formed within the tube network to the same degree (Fig. 3D) in normoxia and severe hypoxia in the Matrigel™ assay (Fig. 3C). Finally, the sprout area was reduced to the same extent (Fig. 3F) in the hanging drop assay (Fig. 3E). Severe hypoxia did not change either the above functions under control conditions.

### Severe hypoxia confers resistance to the anti-proliferative action of vinorelbine

We further addressed whether hypoxia mediates resistance to the anti-angiogenic action of vinorelbine by examining the effect of severe hypoxia (0.1% O_2_) on endothelial cell proliferation (19).

**Figure 4:**
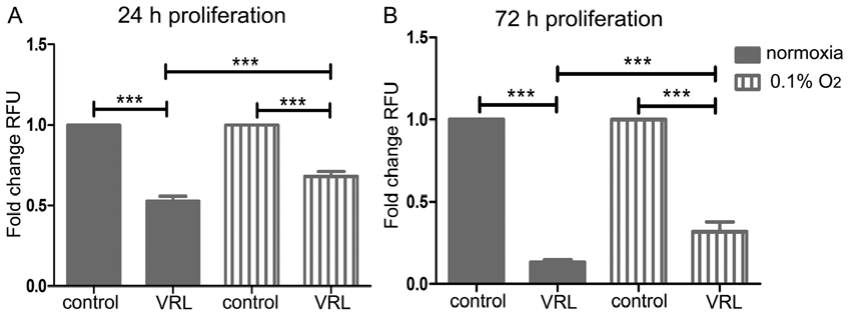
The effect of severe hypoxia (0.1% O_2_) on proliferation of HUVECs treated with metronomic vinorelbine (VRL) as determined by the CyQUANT assay. (A) The effect of severe hypoxia after treatment with 10 nM VRL for 24 h. (B) The effect of severe hypoxia after treatment with 10 nM VRL for 72 h. Proliferation (y-axis) is assessed by the relative fluorescent unit (RFU) normalized to the corresponding control in normoxia or severe hypoxia. Results are expressed as mean ± SD of three independent experiments. Error bars denote standard deviation (SD). One-way ANOVA; ns, not significant; ^***^P<0.001.

After 24-h treatment, 10 nM vinorelbine inhibited proliferation more potently in normoxia than in severe hypoxia (47% versus 32%, P<0.001) (Fig. 4A). Likewise, after 72-h treatment, 10 nM vinorelbine inhibited proliferation to a greater extent in normoxia than in severe hypoxia (87% versus 68%, P<0.001) (Fig. 4B).

### Resistance to the anti-proliferative action of metronomic vinorelbine is due to attenuation of the mitotic arrest and protection from apoptosis

To elaborate the mechanism of resistance to the anti-proliferative action of metronomic vinorelbine, we examined the effect of severe hypoxia on the cell cycle and apoptosis.

**Figure 5:**
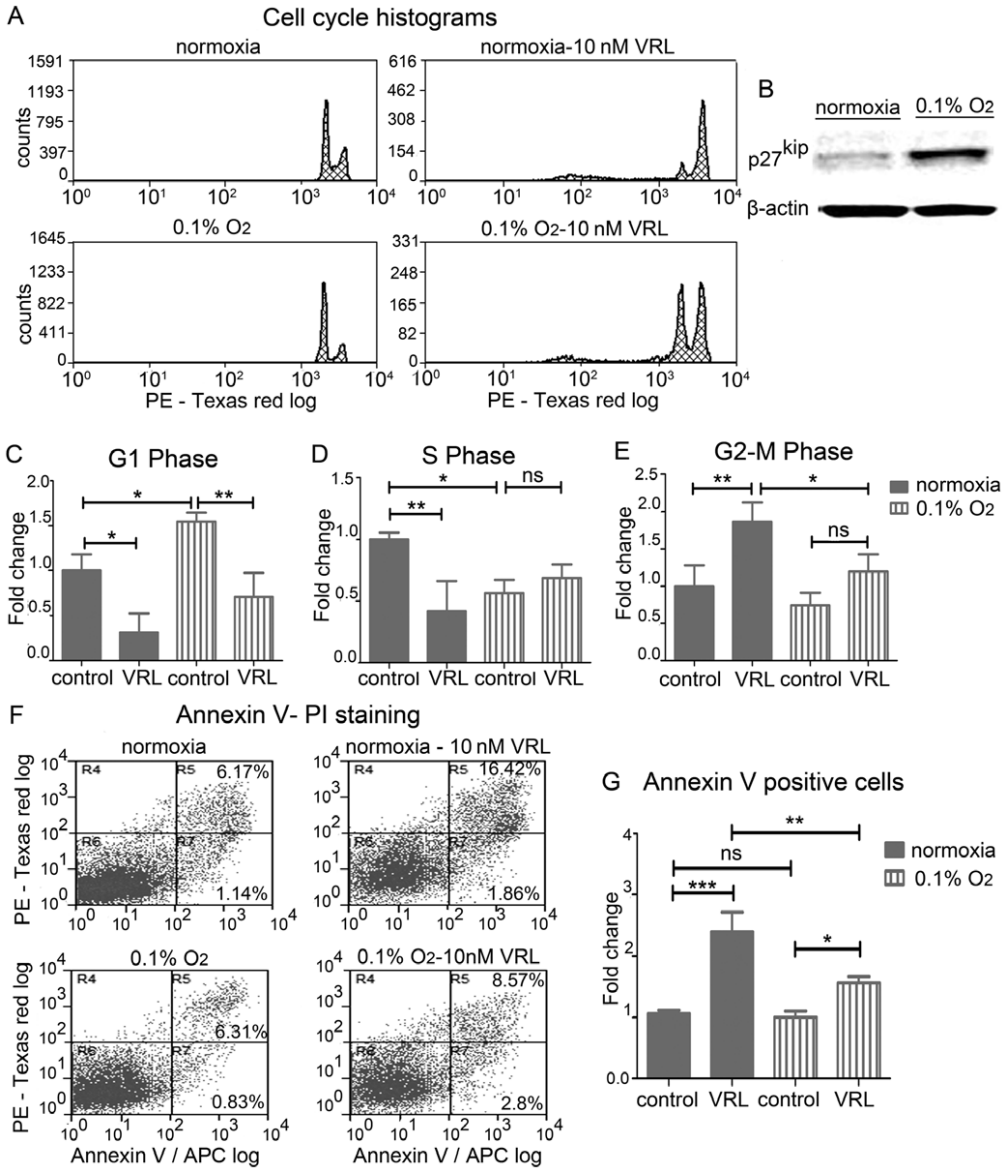
The effect of severe hypoxia (0.1% O_2_) on the cell cycle and apoptosis of HUVECs treated with the metronomic concentration of vinorelbine (VRL). (A) Representative histograms from cell cycle analysis with FACS showing redistribution of the cell cycle phases induced by VRL and severe hypoxia. (B) Western blotting showing upregulation of p27^kip^ in severe hypoxia. (C) Severe hypoxia increases the proportion of cells being in the G1 phase. (D) Severe hypoxia diminishes the cell population in the DNA synthesis S phase. (E) Severe hypoxia attenuates the G2/M arrest induced by VRL. (F) Representative dual parametric dot plot showing staining for Annexin V and/or PI. The values represent percentages of the total cell number. R4, PI-positive (necrotic cells); R5, Annexin V and PI-positive (late apoptotic cells); R6, negative (viable cells); R7, Annexin V-positive (early apoptotic cells). R5+R7, total apoptotic cells. (G) The effect of VRL and severe hypoxia on apoptosis as determined by the Annexin V-positive cells. Results are expressed as mean fold change ± SD of three independent experiments. Error bars represent standard deviation (SD). One-way ANOVA; ns, not significant; ^*^P<0.05; ^**^P<0.01; ^***^P<0.001.

We hypothesized that severe hypoxia slows down proliferation and alters the cell cycle, making vinorelbine less effective in targeting mitotic microtubules (20). We show that severe hypoxia for 24 h upregulated the cyclin-dependent kinase (cdk) inhibitor p27^kip^ (Fig. 5B), which is suggested to block G1/S transition and results in G1 arrest (21). Cell cycle analysis revealed alterations of the distribution of HUVECs in the different cell cycle phases (Fig. 5A). In particular, severe hypoxia increased the percentage of HUVECs in the G1 phase by 1.55-fold (P<0.05) (Fig. 5C) causing a G1 phase arrest while it concomitantly decreased the fraction of the cells in the DNA synthesis S phase by 1.78-fold (P<0.05) (Fig. 5D). Metronomic vinorelbine induced G2/M arrest in normoxia but severe hypoxia attenuated this effect by 1.56-fold (P<0.05) (Fig. 5E).

We next questioned whether the effect of severe hypoxia on the cell cycle leads to apoptotic cell death. Annexin V staining and FACS (Fig. 5F) after 36 h of hypoxia revealed no change in apoptosis levels in untreated cells, however, hypoxia decreased the effect of vinorelbine-induced apoptosis by 1.53-fold (P<0.01) (Fig. 5G).

### Ten nanomolar vinorelbine fails to regulate the Bcl-2/Bax ratio in severe hypoxia

**Figure 6:**
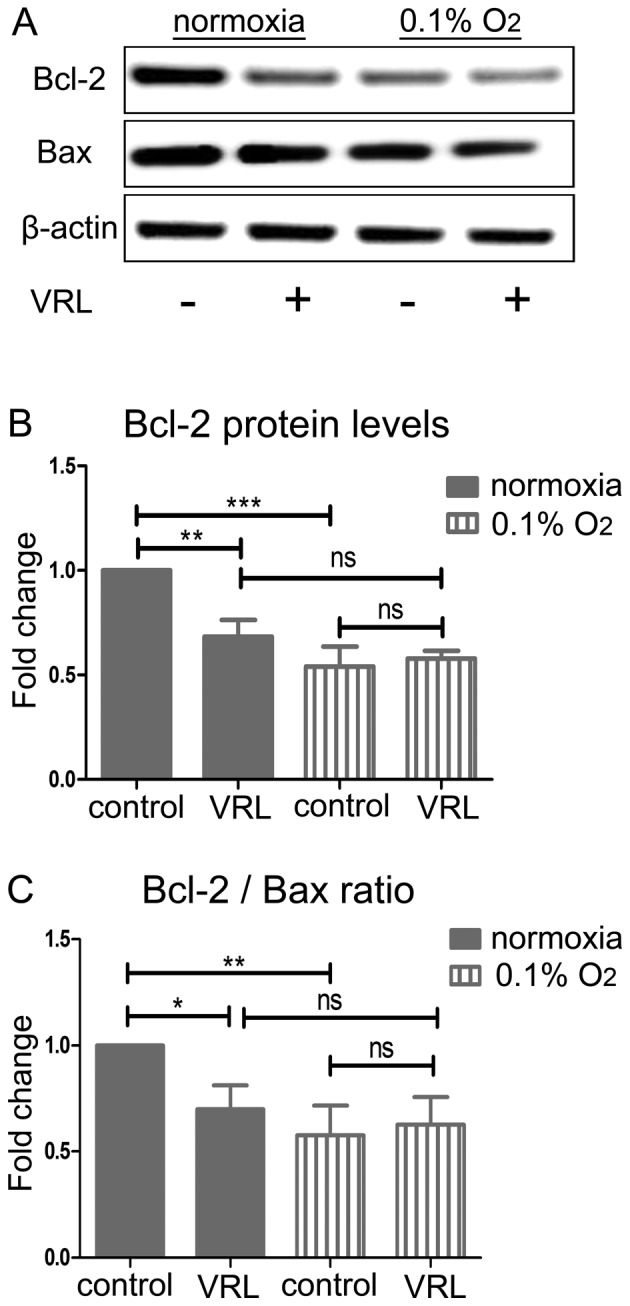
Bcl-2 protein downregulation by 10 nM vinorelbine (VRL) and severe hypoxia (0.1% O_2_). (A) Representative western blotting for Bcl-2 and Bax. (B) The effect on Bcl-2 protein levels. (C) The effect on Bcl-2/Bax ratio. Results are expressed as mean fold change ± SD of three independent experiments. Error bars depict standard deviation (SD). One-way ANOVA; ns, not significant; ^*^P<0.05; ^**^P<0.01; ^***^P<0.001.

To determine the mechanism of protection from the pro-apoptotic action of metronomic vinorelbine we investigated the balance of the anti-apoptotic Bcl-2 and pro-apoptotic protein Bax (Fig. 6A). Bcl-2 and Bax are players of the intrinsic mitochondrial apoptotic pathway and a low Bcl-2/Bax ratio leads to apoptotic cell death through mitochondrial outer membrane permeabilization (22) (MOMP). Moreover, Bcl-2 downregulation has previously been implicated in the cell death caused by vinorelbine (23). Ten nanomolar vinorelbine downregulated the anti-apoptotic protein Bcl-2 in normoxia by 32% (P<0.01) at 24 h. Severe hypoxia also decreased Bcl-2 protein by 46% (P<0.001) but 10 nM vinorelbine did not further reduce Bcl-2 under these conditions (Fig. 6B). Similar changes were seen in the Bcl-2/Bax ratio (Fig. 6C). In particular, 10 nM vinorelbine decreased the Bcl-2/Bax ratio by 30% (P<0.05) in normoxia at 24 h, which is consistent with induction of apoptosis. Severe hypoxia decreased the Bcl-2/Bax ratio by 42% (P<0.01) while 10 nM vinorelbine did not have an additional effect.

### Akt inhibition sensitizes hypoxic endothelial cells to the anti-proliferative and pro-apoptotic action of metronomic vinorelbine

To circumvent the counterproductive effect of severe hypoxia, we examined Akt inhibition as a possible means to reverse hypoxic resistance *in vitro*.

**Figure 7:**
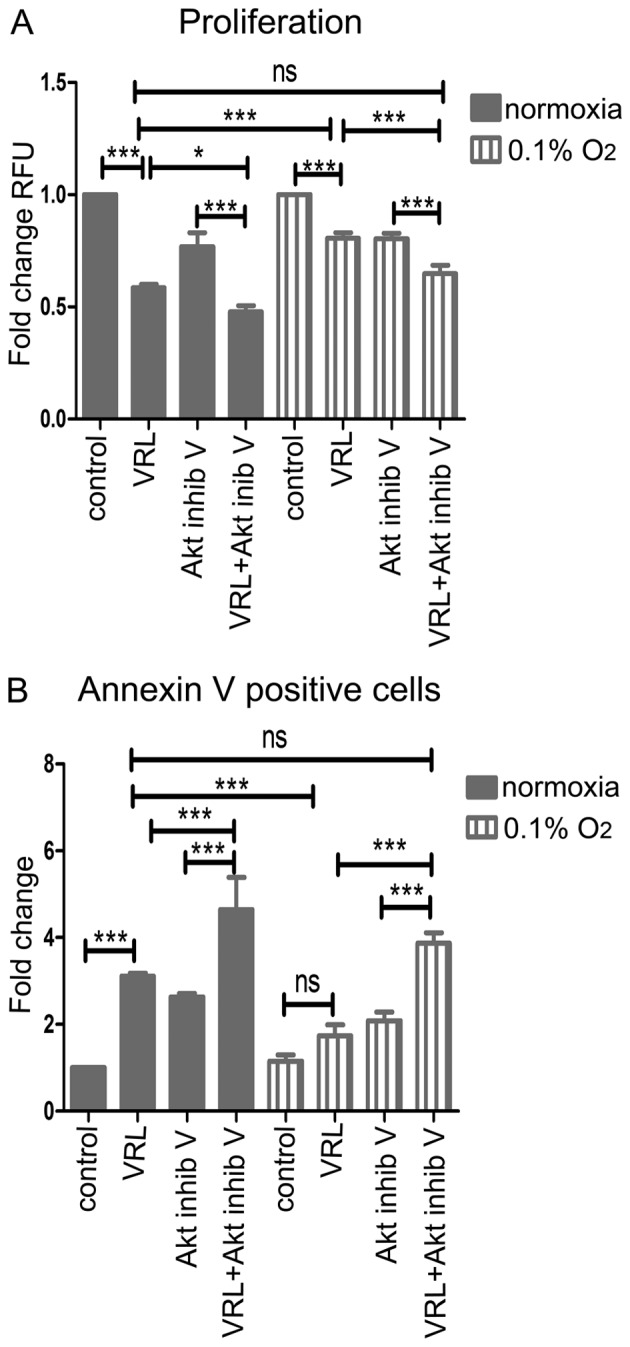
Reversal of the hypoxic resistance by Akt inhibition. (A) The effect of the Akt inhibitor V (Akt inhib V) on proliferation of HUVECs treated with 10 nM vinorelbine (VRL) in normoxia or severe hypoxia (0.1% O_2_) for 24 h. Proliferation was estimated by the relative fluorescence unit (RFU) normalized to the corresponding untreated control in normoxia or severe hypoxia. (B) The effect of the Akt inhibitor V on apoptosis of HUVECs treated with 10 nM VRL in normoxia or severe hypoxia for 36 h as determined by the Annexin V-positive cells. Results are expressed as mean fold change ± SD of three independent experiments. Error bars denote standard deviation (SD). One-way ANOVA; ns, not significant; ^*^P<0.05; ^***^P<0.001. The cells were pretreated with 10 μM of the Akt inhibitor V for 1 h prior to addition of VRL.

Akt inhibition increased the anti-proliferative effect of metronomic vinorelbine in severe hypoxia (Fig. 7A). In particular, 10 nM vinorelbine plus Akt inhibitor V inhibited proliferation more potently than 10 nM vinorelbine alone (35% versus 20% inhibition, P<0.001) after 24-h treatment. Moreover, Akt inhibition in severe hypoxia restored the anti-proliferative effect of vinorelbine to normoxic levels.

Finally, Akt inhibition increased the pro-apoptotic effect of metronomic vinorelbine in severe hypoxia (Fig. 7B). Ten nanomolar vinorelbine plus Akt inhibitor V were more effective, by 2.48-fold (P<0.001), compared to 10 nM vinorelbine alone in inducing apoptosis after 36-h treatment. Furthermore, Akt inhibition in severe hypoxia restored the pro-apoptotic effect of vinorelbine to normoxic levels.

## Discussion

In this study, we demonstrated that the clinically relevant metronomic concentration of vinorelbine, determined in previous clinical trials (4,5), inhibited endothelial cell proliferation. The prolonged treatment with 10 nM metronomic vinorelbine was superior to the short-term treatment with 1 μM. Given that 1 μM vinorelbine approximates the transient peak plasma levels of the drug in pharmacokinetic studies of conventional chemotherapy (18), the *in vitro* short exposure to 1 μM could simulate the pulsatile administration of a maximum tolerated dose. The fact that a treatment that simulates the chronic low dose chemotherapy *in vitro* had greater effect than a treatment that resembles conventional chemotherapy advocates the use of vinorelbine in a metronomic regimen. These results are in line with other studies which denote that endothelial cells are more sensitive to metronomic than conventional chemotherapy. Bertolini *et al* demonstrated that the viability of circulating endothelial progenitors (CEP) in mice is reduced upon treatment with metronomic cyclophosphamide while treatment with the maximum tolerated dose increased their number during the drug-free periods (24). Pasquier *et al* reported that immortalized endothelial cells have impaired ability to form vascular structures and increased sensitivity to chemotherapy after continuous treatment with non-toxic concentrations of vinblastine as opposed to prior treatment with a maximum tolerated concentration (25).

Furthermore, we proved that the metronomic concentration of vinorelbine is anti-angiogenic *in vitro*. We showed that metronomic vinorelbine inhibited critical events of the angiogenic process in a complete array of angiogenic assays. Apart from endothelial cell proliferation, we demonstrated that metronomic vinorelbine inhibited migration and tube formation whereas the conventional concentration of 1 μM (18) inhibited these functions with simultaneous induction of cell death. Moreover, we showed that metronomic vinorelbine inhibited endothelial cell sprouting. Our results agree with accumulated evidence regarding microtubule targeting agents (MTAs) (26). Vinflunine was shown to be anti-angiogenic *in vitro* at concentrations that do not affect proliferation (27) and paclitaxel was shown to inhibit functions of the endothelial cell biology at ultra low concentrations (28). Our *in vitro* evidence on the anti-angiogenic activity of metronomic vinorelbine is in concert with the clinical evidence provided by Briasoulis *et al* (4,5), where patients that responded to metronomic vinorelbine treatment expressed low levels of circulating pro-angiogenic biomarkers, whereas patients that failed to respond expressed markers associated with resistance to anti-angiogenesis.

Despite promising preclinical data, clinical practice has shown that even responding patients become eventually refractory to anti-angiogenic therapy (7). Treatment-induced hypoxia emerges as a major mechanism of resistance to anti-angiogenic therapy (8). We found that vinorelbine inhibited migration, tube formation and sprouting to the same extent in normoxia and severe hypoxia. Interestingly, severe hypoxia did not have any effect *per se* on these functions. The latter may be explained by the fact that we cultivated HUVECs in media supplemented with growth factors and fetal bovine serum (FBS). Calvani *et al* demonstrated that hypoxia enhanced formation of tube-like structures when they cultured HUVECs in media depleted from growth factors due to the autocrine action of hypoxia induced b-FGF (29). We did cultivate HUVECs in growth media to simulate the complex tumor microenvironment (6) rather than a condition-dependent on b-FGF alone. Similarly, Calvani *et al* showed that tube formation in hypoxia is comparable to that in normoxia when HUVECs were challenged with growth factor containing media (29).

However, we found that severe hypoxia mediated resistance to the anti-proliferative action of vinorelbine. This in line with observations from other investigators who showed that hypoxia mediates resistance to the anti-proliferative action of chemotherapeutics in cancer cells (10,11). We demonstrated that severe hypoxia induces G1 arrest in HUVECs. This is consistent with the upregulation of the cyclin-dependent kinase (cdk) inhibitor p27^kip^ that we detected. Hypoxia is suggested to increase p27^kip^ protein which arrests cells in G1 phase by inhibiting CDK2 activity which prevents entry to S phase as described by Gardner *et al* (21). Vinca alkaloids are suggested to exert their activity by blocking mitosis and arresting cells in G2/M phase (20). In particular, vinorelbine suppresses microtubule dynamics and thus disorganizes the mitotic spindle which fails to congress chromosomes during mitosis, as reported by Ngan *et al* (30). The perturbation of this process blocks the metaphase to anaphase transition (30). In accordance with this, we found that metronomic vinorelbine induced G2/M arrest in endothelial cells. However, we show that severe hypoxia attenuated the G2/M block as it shifted the cells in G1 phase where they are insensitive to vinorelbine which is a cell cycle specific agent (20). We therefore propose that severe hypoxia interferes with the action of metronomic vinorelbine in blocking mitosis.

Besides altering the cell cycle, we demonstrated that severe hypoxia lessened the pro-apoptotic action of metronomic vinorelbine as determined with Annexin V staining. Activation of the mitochondrial intrinsic pathway and downregulation of the Bcl-2 protein is suggested to be the mechanism of cell killing by vinorelbine (23). Specifically, the balance between the pro-apoptotic and anti-apoptotic proteins of the Bcl-2 family dictates whether the cell undergoes apoptosis or not (22). Consequently, we examined the levels of the pro-apoptotic Bax and anti-apoptotic Bcl-2 which are regulated by vinca alkaloids.

We show that metronomic vinorelbine decreased the Bcl-2 protein and the Bcl-2/Bax ratio in normoxia that is consistent with induction of apoptosis. Severe hypoxia decreased the Bcl-2 protein and the Bcl-2/Bax ratio. Treatment with metronomic vinorelbine did not further reduce the ratio compared to the hypoxic control. Therefore, vinorelbine failed to regulate the Bcl-2 protein in severe hypoxia as opposed to normoxia and this may account for its decreased pro-apoptotic action. Although Bcl-2 is anti-apoptotic, some authors suggest that the low Bcl-2 levels correlate with poor response to microtubule targeting agents. Esteve *et al* found that downregulation of Bcl-2 is associated with resistance of ovarian cancer cells to vinflunine (31). Moreover, Savry *et al* demonstrated that the Bcl-2 overexpression enhances the efficacy of vinorelbine and paclitaxel in lung and breast cancer cells through upregulation of Bim (32). Therefore, we speculate that the low Bcl-2 protein levels in hypoxic endothelial cells may contribute to poor efficacy of vinorelbine in severe hypoxia.

We sought to find a way to overcome the resistance in severe hypoxia. We found that the addition of the Akt inhibitor V increased the sensitivity to the anti-proliferative effect of vinorelbine in severe hypoxia. Moreover, Akt inhibition enhanced the pro-apoptotic action of vinorelbine in normoxia and severe hypoxia and restored the effect of vinorelbine in severe hypoxia to normoxic levels.

The Akt pathway mediates cell survival through multiple mechanisms (15). In particular, it inhibits directly caspase 3 and 9 (33), the pro-apoptotic proteins Bad and Bax (34) as well as GSK3β (35). Akt suppresses functionality of the pro-apoptotic proteins Bad and Bax, rendering them unable to permeabilize mitochondrial membrane. Furthermore, Akt inhibition leads to GSK3β activation and subsequently disruption of hexokinase II (HK II) binding from the mitochondrial membrane (36). Dissociation of HK II triggers apoptosis through mitochondrial permeability transition (MPT) (37) which is a distinct mechanism of mitochondrial permeabilization that allows mitochondrial swelling, outer membrane disruption and cytochrome *c* (38) release independently of the presence of Bax and Bak (35). Finally, Akt inactivates caspase 3 and 9 by a posttranslational modification (33). The caspase cascade is the final step of apoptosis where breakdown of the cell takes place. Hence, Akt inhibition seems to be a reasonable way to enhance the sensitivity to apoptotic stimuli as it acts in multiple levels of the apoptotic process.

In conclusion, we report that the clinically relevant metronomic concentration of 10 nM vinorelbine is anti-angiogenic *in vitro* and we speculate that its clinical efficacy can be attributed at least in part to anti-angiogenesis. Severe hypoxia, which is potentially induced by anti-angiogenic treatment, has a counterproductive effect and can be a factor of treatment failure. It confers resistance to its anti-proliferative action by modulating cell cycle and apoptotic cell death. Akt inhibition appears to be a promising target for combination in order to circumvent hypoxic resistance and warrants further investigation.
